# Analysis of oxygen consumption rates in zebrafish reveals differences based on sex, age and physical activity recovery

**DOI:** 10.3389/fphys.2023.1272366

**Published:** 2023-09-13

**Authors:** Bridget Konadu, Jonathan P. Hosler, Yann Gibert, Kristin S. Edwards

**Affiliations:** ^1^ Department of Cell and Molecular Biology, Cancer Center and Research Institute, University of Mississippi Medical Center, Jackson, MS, United States; ^2^ Department of Pharmacology, University of Mississippi Medical Center, Jackson, MS, United States

**Keywords:** zebrafish, oxygen consumption, ageing, sex differences, embryogenesis, physical activity

## Abstract

**Introduction:** Mitochondrial dysfunction is linked to a variety of human diseases. Understanding the dynamic alterations in mitochondrial respiration at various stages of development is important to our understanding of disease progression. Zebrafish provide a system for investigating mitochondrial function and alterations during different life stages. The purpose of this study was to investigate our ability to measure mitochondrial oxygen consumption rates in zebrafish embryos, larvae, and adults as an indicator of mitochondrial function.

**Methods:** Basal respiration of entire zebrafish embryos (5 dpf), larvae (0.6–0.9 cm), young adults (3-month-old), and old adults (12-month-old) was measured using an Oroboros Oxygraph, with a stirrer speed of 26 rpm. For embryos and larvae, “leak” respiration (plus oligomycin), maximum respiration (plus uncoupler), non-mitochondrial respiration (plus inhibitors), and complex IV activity were also measured. To induce physical activity in adult fish, the stirrer speed was increased to 200 rpm.

**Results and Discussion:** We demonstrate the ability to accurately measure respiration rates in zebrafish at various ages using the Oroboros Oxygraph. When comparing zebrafish embryos to larvae, embryos have a higher maximum respiration. Three-month-old zebrafish males have higher basal respiration than females, while 12-month-old zebrafish females exhibit greater rates of respiration than males and younger females. When the stirrer speed was increased, respiration rates decrease, but with differences depending on sex. This study demonstrates a simple and accessible method to assess zebrafish physiology by mitochondrial oxygen consumption measurements in an unmodified Oroboros Oxygraph. The method should facilitate studies to understand the intricate interplay between mitochondrial function, development, and aging.

## 1 Introduction

Mitochondrial function is critical for energy metabolism and cell homeostasis (Mishra and Chan, 2016). Mitochondrial dysfunction has been linked to a variety of human diseases, including abnormalities in metabolism, neurological conditions, and aging (Wallace, 2011; Wang et al., 2019; San-Millán, 2023). Understanding the dynamic alterations in mitochondrial respiration at various stages of development is therefore critical for elucidating the complex interplay between mitochondrial function and organismal physiology.

Understanding cellular energy metabolism and its implications for various physiological processes requires quantifying various components of mitochondrial respiration (Chen et al., 2016). Measurements of cellular or organismal respiration provide a global analysis of mitochondrial function. Typical cellular or organismal respiration assays include basal or resting respiration, which can report how mitochondrial activity changes with development, genetic alterations, *etc.*, respiration in the absence of ATP synthase activity, which reports changes in the rate of passive proton leak across the mitochondrial inner membrane and maximum respiration in the presence of uncoupler, which reports if substrate oxidation and mitochondrial electron transfer from NADH to O_2_ are occurring at normal rates compared to a control. In an assay, that is, often overlooked, the activity of the Complex IV and the soluble cytochrome *c* pool can also be measured by cellular O_2_ consumption by supplying electrons to cytochrome c in the inner membrane space of mitochondria using hydrophobic electron carriers. Changes in the rate of complex IV activity may indicate changes in the amount of inner membrane in the cell. Moreover, the rate of Complex IV O_2_ consumption in the presence of oligomycin or uncoupler provides information about passive proton leak and the response of a proton pump to the membrane potential that can be compared to similar probing of the entire respiratory system outlined above (Brand and Nicholls, 2011).

The Oroboros Oxygraphs/Fluorespirometers are highly popular tools for high-resolution respirometry of purified enzymes, such as complex IV, isolated mitochondria, tissue slices and homogenates and intact cells (Long et al., 2019). A key feature of the Oroboros system is extremely low background noise, which allows for accurate measurements of O_2_ consumption rates over time. The dual 2 mL glass chambers, each with their own O_2_ electrode, can hold up to sixty zebrafish embryos, five larvae, one 3-month adults and one 12-month adults (Bioblast, 2002).

The zebrafish (*Danio rerio*) has emerged as an effective model organism for investigating various biological processes due to its genetic tractability, optical transparency, and flexibility to experimental manipulation (Teame et al., 2019). Furthermore, zebrafish mitochondria are highly similar to mammalian mitochondria, making them a promising system for investigating mitochondrial function and its modulation during different life stages. To date, most studies on zebrafish mitochondrial respiration have predominantly focused on specific developmental periods or adult stages. However, a comprehensive comparative analysis encompassing key developmental stages, including embryos, larvae, young adults, and older adults, has not yet been examined. A study like this could provide vital insights into the dynamics of mitochondrial respiration at different stages of life and shed light on age-dependent changes in energy metabolism and mitochondrial function. In this study, we used the malleability of the zebrafish to adapt to the Oroboros chamber at different stages of life, and we used a novel approach to examine the developmental stage-specific variation in oxygen consumption rates, as well as the sex-specific changes in zebrafish mitochondria at different life stages. This study will contribute to our understanding of the intricate interplay between mitochondrial function, development, and aging, with implications for both zebrafish research and our broader understanding of vertebrate mitochondrial physiology.

## 2 Materials and methods

### 2.1 Animal

All animal husbandry and experimental techniques were reviewed and approved by the UMMC institutional animal care and use committee (IACUC #1551). The zebrafish were handled in accordance with the specified guidelines of the IACUC. Embryos were generated using an AB wildtype strain received from the Zebrafish International Resource Center (ZIRC) and raised in embryonic medium (E3) at 28.5°C under standard conditions with a 14 h light/10 h dark cycle. Zebrafish embryos that were 5 dpf were used to measure mitochondria respiration. Larvae were raised on normal zebrafish diet to 0.6–0.9 cm long as well as young adult 3 months old and old adult 12 months of age. Larvae are defined as zebrafish that are 0.6–0.9 cm long, where length is measured from the tip of the snout to the posterior point of the notochord (a flexible rod-like structure) (Parichy et al., 2009). This method compensates for changes in body curvature and thus provides a more accurate representation of the fish size.

### 2.2 Experimental protocol

**FIGURE 1:**
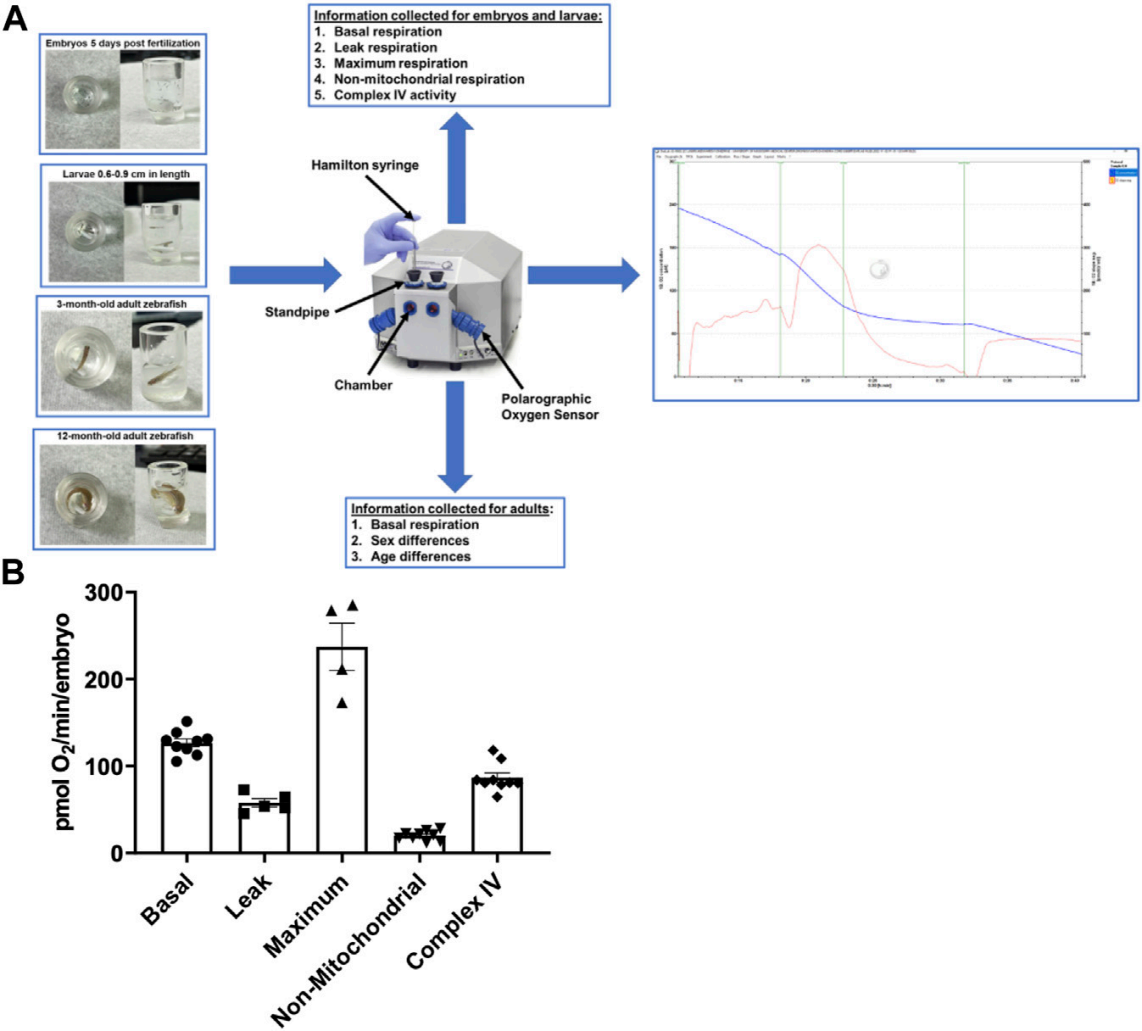
**(A)** Representation of the zebrafish in the chambers, the Oroboros Oxygraph, and the information output for each age of the zebrafish. The zebrafish are placed in the Oroboros oxygraph chamber with 2 mL of E3 media with the stirrer speed set to 26 rpm. The chambers are closed to accurately measure the oxygen concentration in the chamber. If additional reagents are added, they are added through the standpipes via a Hamilton syringe. An example of the output in embryos is on the right side of the figure. The blue line is oxygen concentration in the chamber. The red line is the tangent of the blue line. **(B)** Mitochondrial respiration measurements in 5 days post fertilization embryos using the Oroboros stir bar set at a speed of 26 rpm. Basal respiration was measured 5 min after the embryos acclimated to the chamber (circles). Oligomycin was used to initiate leak respiration (25 μM; squares). Maximum respiration was achieved by adding FCCP (2.5 µM, triangles). Rotenone (1.3 µM) and antimycin A (1.8 µM) were used to determine non-mitochondrial respiration (upside down triangles). Complex IV activity (diamonds) was measured by the addition of ascorbate (10 mM) and TMPD (0.3 mM). Each point equates to one experiment and each experiment contains 60 embryos.

The Oroboros Oxygraph was used to measure respiration rates in zebrafish (Figure 1A). Zebrafish were used at the following ages: embryos at 5 days post fertilization, larvae sized 0.6–0.9 cm long, 3-month-old adults, and 12-month-old adults old. For each experiment, sixty embryos, five larvae, or one young or old adult were used. Zebrafish embryos are so tiny that their individual oxygen consumption in the embryonic medium is very difficult to assess. To get around this issue, we pooled 60 embryos per experiment. While larvae are larger we still required 5 larvae per experiment. Therefore, for each experiment, sixty embryos, five larvae, or one young or old adult were used (n denotes the number of experiments performed). For each experiment, basal, non-mitochondrial, and complex IV respiration rates were measured. As a result, we have 4–5 oligomycin (leak) experiments and 4–5 FCCP (maximum) experiments. Before being placed in the chamber with 2 mL of zebrafish E3 experimental medium, zebrafish were washed in experimental media. For measurements with the embryos, the provided stir bar from Oroboros was used. For larvae and adult zebrafish, a thinner stir bar (FisherbrandTM Octagon SpinbarTM Magnetic Stirring Bars Cat # 14-513-98) was used. Stirrer speeds were set to 26 rpm unless otherwise noted. We chose this speed because faster speeds have the potential to homogenizer the embryos. All respiration rates were allowed to stabilize for 5 min. The temperature was set to 28°C. A calibration for the oxygen concentration was performed for each experiment. Oxygen consumption rates were normalized to the number embryos and larvae.

### 2.3 Respiration measurements in embryos, larvae, and adult zebrafish

Once embryos or larvae were in the chamber, basal respiration rates were allowed to stabilize for 5 min. To determine the leak respiration rate 25 µM oligomycin, a selective inhibitor of the mitochondrial ATP synthase, was injected into the chamber. The rate was allowed to stabilize for 5 min. To determine the non-mitochondrial respiration rate, 1.8 µM antimycin A, an inhibitor of complex III of electron transport chain, 1.3 µM rotenone, an inhibitor of complex I of electron transport, was added and the rate was allowed to stabilize for 5 min. Complex IV activity was measured by adding 10 mM ascorbate and 0.3 mM N, N, N′, N′-tetramethyl-p-phenylenediamine (TMPD). In a separate experiment, the maximum respiration rate was measured by adding the uncoupler FCCP (carbonyl cyanide-p-trifluoromethoxyphenylhydrazone) at a concentration of 2.5 µM for embryos and in 5.0 µM for larvae. This was done to obtain a more accurate measure of the maximum respiration rate. Non-mitochondrial respiration and complex IV activity was measured as described above. To compare the respiration rates between embryos and larvae, a ratio of each respiration measurement was normalized to the initial basal respiration for the experiment. In adult zebrafish, basal respiration was only measured due to the fast rate of oxygen consumption. Therefore, when comparing age and sex in the adult zebrafish only basal respiration rates were used.

### 2.4 Respiration rates in response to stirrer speeds in young adult zebrafish

To assess activity levels in both males and females at 3 months of age, the zebrafish were subjected to stirrer speeds of 26, 100, and 200 rpm consecutively before returning to 26 rpm. At each speed the respiration rate was allowed to stabilize for 5 min before changing the speed. The return to 26 rpm allowed for the comparison from initial rate of respiration to the rate after exercise.

### 2.5 Statistical analysis

Data are expressed as mean ± SEM. Comparisons between two groups were performed using unpaired Student’s *t*-test. Comparisons between multiple experimental groups were made by ordinary two-way ANOVA analysis with uncorrected Fisher’s LSD, with a singled pooled variance. A *p*-value <0.05 was considered statistically significant. All the analyses were performed with GraphPad Prism version 9.

## 3 Results

### 3.1 Oxygen consumption measurements in zebrafish embryos and larvae

The Oroboros system was used to examine several aspects that contribute to overall cellular respiration and ATP production in order to estimate mitochondrial function and efficiency (Figure 1A). All measurements were taken at the same stir speed to ensure optimal oxygen distribution and mixing within the oroboros chamber. In 5 days post fertilization (dpf) embryos (*n* = 60), basal oxygen consumption, which evaluates baseline fundamental metabolic activity without the use of any reagent, was examined. Embryos were exposed to a minimum stirrer speed of 26 rpm in the Oroboros chamber, and our data reveal that embryos consume 126 ± 4.6 pmol O_2_ per minute per embryo (Figure 1B). Proton leak is a mitochondrial characteristic that can affect energy metabolism and mitochondrial efficiency (Jastroch et al., 2010). Therefore, in the presence of oligomycin, a specific inhibitor of the mitochondrial ATP synthase, the leak respiration was 57.7 ± 4.8 pmol O_2_ per minute per embryo (Figure 1B). Furthermore, we assessed maximum respiration when oxidative phosphorylation is uncoupled using FCCP (carbonyl cyanide-p-trifluoromethoxyphenylhydrazone), to calculate the maximal rate of O_2_ consumption based on the electron transfer capacity (Zdrazilova et al., 2022). We determined the maximum respiration rate of for embryos to be 237.1 ± 27.1 pmol O_2_ per minute per embryo (Figure 1B). In addition, the contribution of non-mitochondrial sources to overall oxygen consumption was determined by adding mitochondrial inhibitors rotenone and antimycin A (Pfeiffer et al., 2014). The non-mitochondrial rate was 20.1 ± 5.5 pmol O_2_ per minute per embryo (Figure 1B). Finally, to assess the efficiency for complex IV to catalyze the transfer of electrons to oxygen (Zhao et al., 2019), the respiration rate of Complex IV was determined to be 70 pmol O_2_ per minute for each embryo (Figure 1B).

**FIGURE 2:**
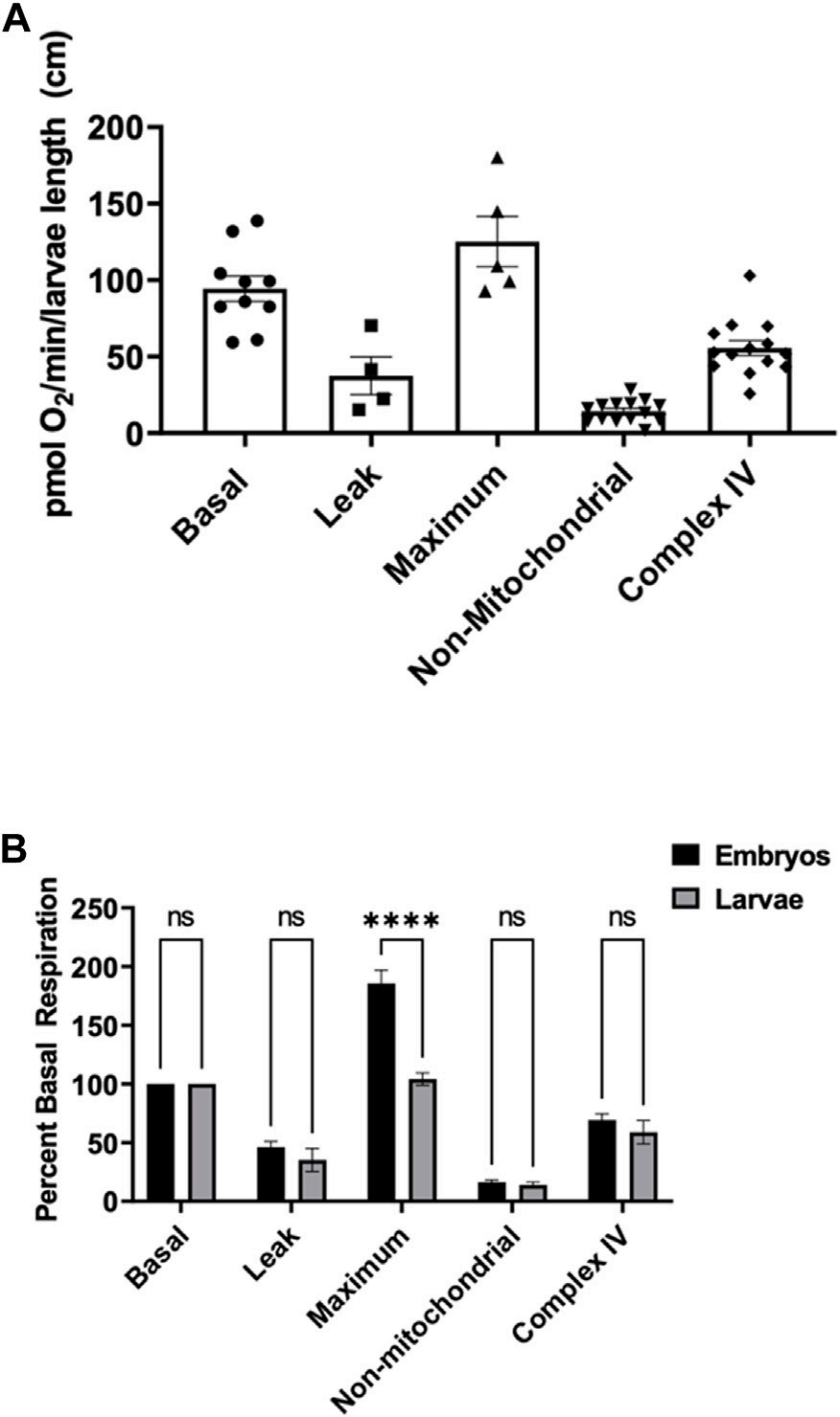
**(A)** Mitochondrial respiration measurements in larvae 0.6–0.9 cm in length using the thinner stir bar (mentioned in methods) set at a speed of 26 rpm. Basal respiration was measured 5 min after the embryos acclimated to the chamber (circles). Oligomycin was used to initiate leak respiration (25 μM; squares). Maximum respiration was achieved by adding FCCP (2.5 µM, triangles). Rotenone (1.3 µM) and antimycin A (1.8 µM) were used to determine non-mitochondrial respiration (upside down triangles). Complex IV activity (diamonds) was measured by the addition of ascorbate (10 mM) and TMPD (0.3 mM). Each point equates to one experiment and each experiment contains 5 larvae ranging in size from 0.6–0.9 cm. **(B)** Comparison of the different respiration rates from Figure 1 and Figure 2 for embryos (black) and larvae (grey). The rates were normalized to the basal respiration rates for each experiment. (**** indicates a *p*-value < 0.00001).

To assess mitochondria efficiency in active larvae, we measured components of mitochondrial respiration as mentioned above in zebrafish embryos. Five larvae were utilized at each point under the same stirrer speed for maximum oxygen distribution in the oroboros chamber. Basal oxygen consumption rate was determined to be 94.4 ± 8.3 pmol O_2_ per minute per larvae (Figure 2A). Exposing larvae to oligomycin, leak respiration rate was determined to be 37.4 ± 12.3 pmol O_2_ per minute per larvae (Figure 2A). The maximum respiration of the mitochondria when exposed to FCCP was determined to be 125.2 ± 16.4 pmol O_2_ per minute per larvae (Figure 2A). Non-mitochondrial respiration in the presence of mitochondrial inhibitors was determined to be 14.3 ± 1.9 pmol O_2_ per minute per larvae and complex IV activity representing efficiency of oxidative phosphorylation was determined to be 55.52 ± 4.9 pmol O_2_ per minute per larvae (Figure 2A).

To compare embryos to larvae, all rates were normalized to the basal respiration rate (Figure 2B). There was no significant difference between proton leak, non-mitochondrial, and complex IV activity when comparing embryos to larvae. However, when oxidative phosphorylation was uncoupled from FCCP, embryos showed a significant higher capacity of maximum mitochondrial respiration than larvae.

### 3.2 Sex differences in basal respiration rates in adult zebrafish

**FIGURE 3:**
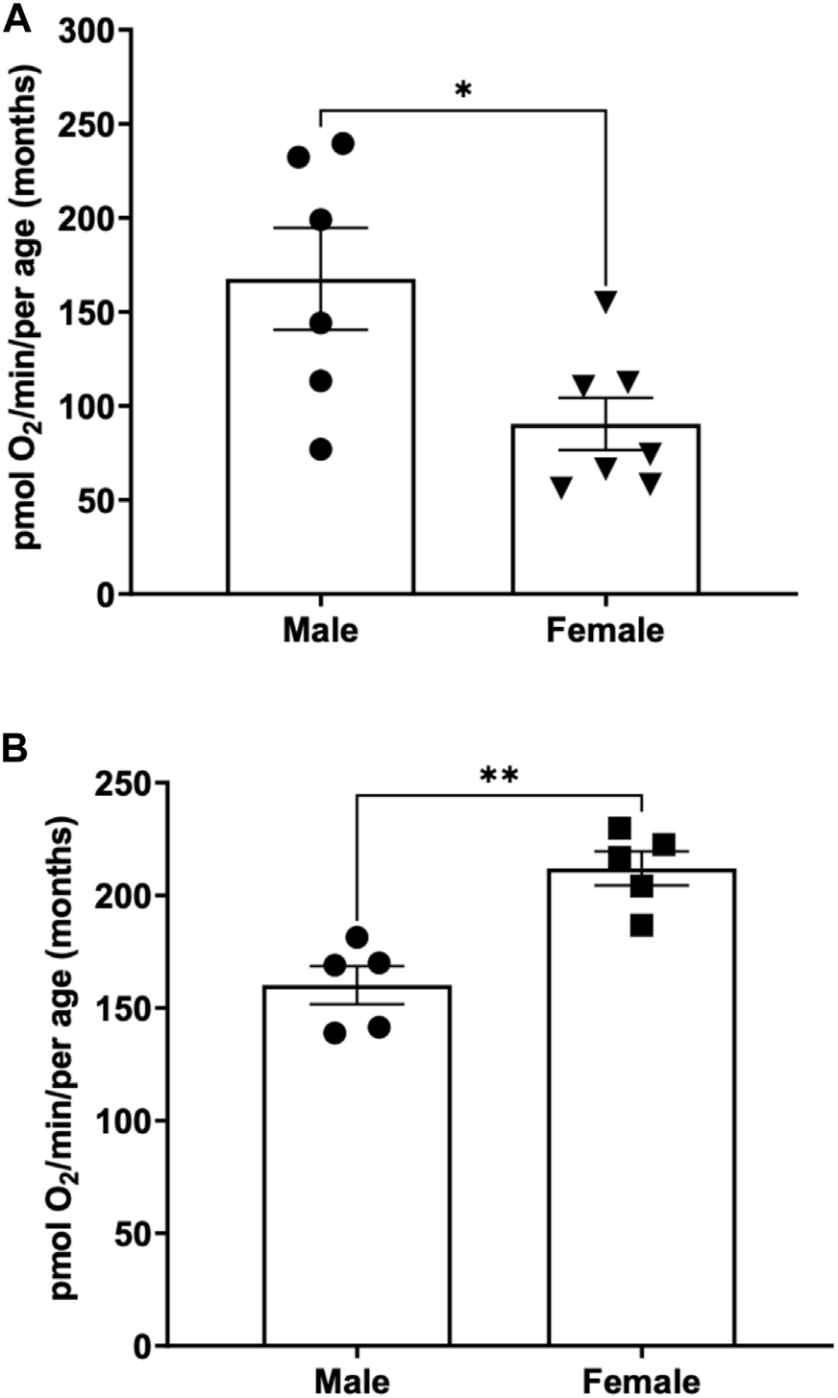
**(A)** Basal respiration rates in young (3-month-old) adult male and female zebrafish. Using the thin stir bar (see methods) set at 26 rpm, basal respiration was measured on males and females. (* indicates a *p*-value < 0.5) **(B)** Basal respiration rates in old (12-month-old) adult male and female zebrafish. Using the thin stir bar (see methods) set at 26 rpm, basal respiration was measured on males and females. (** indicates a *p*-value ≤0.01).

To determine the basal respiration rate in sexually mature adult fish at 3 months of age, both male and females were subjected to 26 rpm stirrer speed and oxygen consumption were measured and compared. We demonstrate that young male adult fish have a significantly higher basal respiration rate (167.6 ± 27.1) pmol O_2_ per minute per adult compared to young female (90.5 ± 13.9) pmol O_2_ per minute per adult that are aged matched (Figure 3A).

To determine whether basal respiration differed between 12-month-old male and female adult zebrafish, males and females aged matched were subjected to a minimum stirrer speed of 26 rpm and oxygen consumption rates were measured. Interestingly, we found the opposite result as the young adults. The older female adult zebrafish showed a significantly higher basal respiration rate (212 ± 7.6) compared to males (160.1 ± 8.4) of the same age (Figure 3B).

### 3.3 Age differences in basal respiration rates in adult zebrafish

**FIGURE 4:**
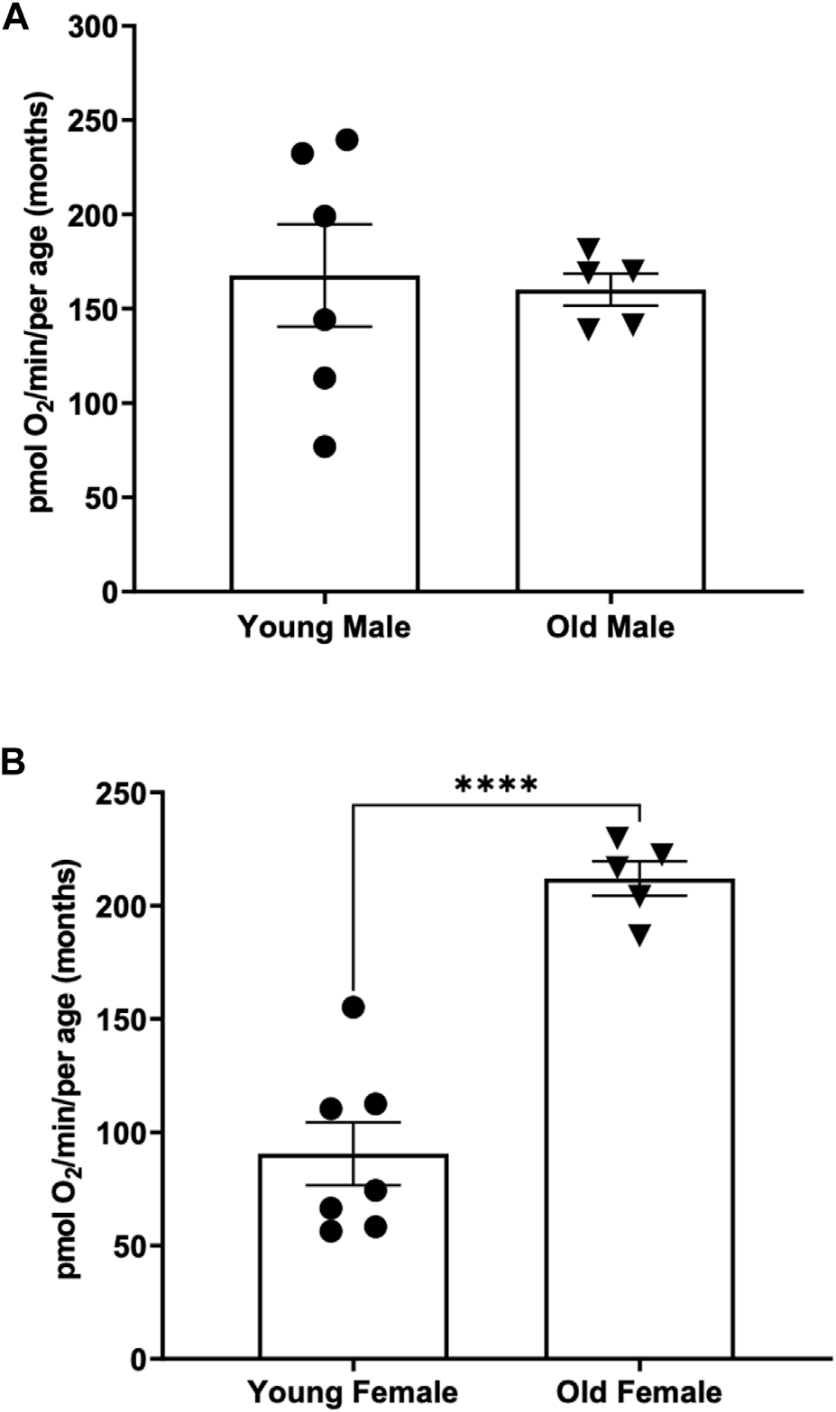
**(A)** Basal respiration rates in young (3-month old) compared to old (12-month-old) adult males. Using the thin stir bar (see methods) set at 26 rpm, basal respiration was measured on males and females. **(B)** Basal respiration rates in young (3-month old) compared to old (12-month-old) adult females. Using the thin stir bar (see methods) set at 26 rpm, basal respiration was measured on males and females. (**** indicates a *p*-value < 0.00001).

When comparing the basal respiration rate of young males (3 months of age) to older males (12 months of age) we found no significant difference between the two (Figure 4A) (167.6 ± 27.13 vs. 160.1 ± 8.45) pmol O_2_ per minute per adult.

Interestingly when comparing the basal respiration rate of young females (3 months of age) to older females (12 months of age) we found that the older female adults have a significantly higher basal respiration rate than the younger females (Figure 4B) (212 ± 7.6 vs. 90.52 ± 13.88) pmol O_2_ per minute per adult.

### 3.4 Alterations to basal respiration rates in response to physical activity

**FIGURE 5:**
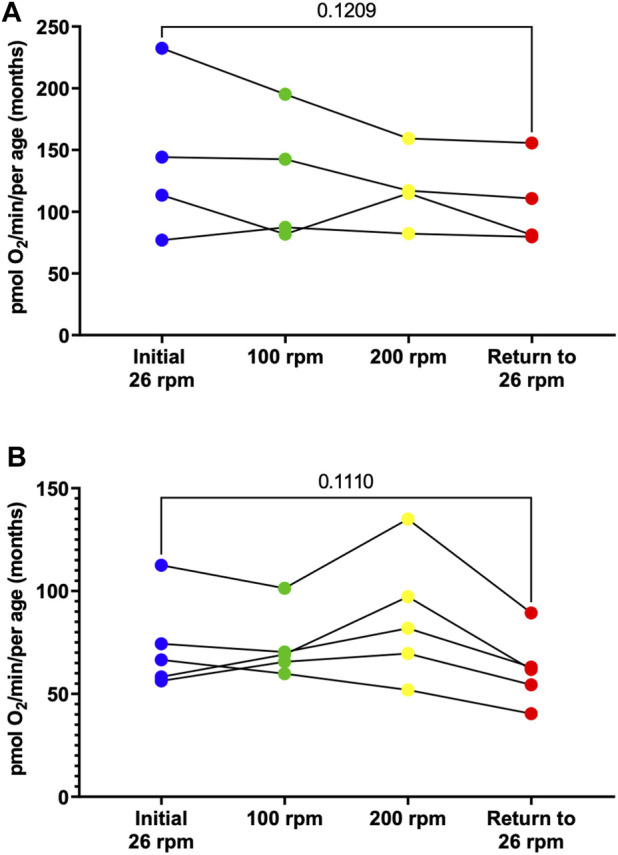
Individual basal respiration rates in young (3-month old) adult males **(A)** and females **(B)**. Using the thin stir bar (see methods) measurements were made initially at 26 rpm (blue), then at 100 rpm (green), then at 200 rpm (yellow), and after return to 26 rpm (red).

To determine whether an increase in activity level alters respiration rates, young (3-month-old) male and female adult zebrafish were subjected to different activity levels corresponding to different stirrer speeds starting at 26 rpm, then increased to 100 rpm for 5 min, 200 rpm for 5 min, and returned to 26 rpm. At various speeds, the rate of oxygen consumption was monitored. While our findings show no significant differences, males show a trend for a decline in respiration in response to activity (Figure 5A, Supplementary Figure S1) while females show a trend for an increase in respiration at 200 rpm that ultimately decreases when returned to 26 rpm (Figure 5B, Supplementary Figure S1). These results demonstrate that using the Oroboros Oxygraph we are able to measure several parameters of mitochondrial respiration in zebrafish embryos, larvae and adult and identify difference in oxygen consumption rates based on the age and the sex of the zebrafish.

## 4 Discussion

This study is the first to explore respiration measurements in zebrafish at ages ranging from 5 days post fertilization to 12-month of age. Previously, zebrafish respiration was measured in the Agilent Seahorse XF24. The Seahorse provides multiwell respirometry for high throughput screening (Zdrazilova et al., 2022). However, changes in reagent additions cannot be made in real time due to the use of a premade cartridge. The wells also limit the movement of the zebrafish. Additionally, the Seahorse XF24 is limited to 5 dpf zebrafish and movement is limited in the well due to a constricting mesh. The Oroboros Oxygraph, used in this study, provides high resolution respirometry with the ability to change reagent additions in real time with the use of Hamilton syringes (Liang et al., 2021). The chamber is larger allowing free movement of the zebrafish and the use of larvae and adult zebrafish. The stir bar allows for movement of the media in the chamber. However, if the stirrer speed is set to the default speed (750 rpm), the zebrafish can become damaged or even homogenized. Therefore, the stirrer speed was set to the minimum setting of 26 rpm and in larvae and adult zebrafish a smaller stir bar was used. Zebrafish embryos flow with the buffer in the chambers. However, beginning at the larvae stage they start to swim. Therefore, the stirrer speed can be increased up to 200 rpm to promote an increase in physical activity of the larvae and adult zebrafish. However, when zebrafish begin to swim respiration measurements are limited to basal respiration rates. This is because reagents, such as FCCP for maximum respiration, are toxic and the fish stop swimming. Therefore, the maximum respiration measured may be lower than the basal respiration rate especially in adult fish. While both machines have their advantages and disadvantages, comparisons of age and sex can be performed more accurately and efficiently in the Oroboros Oxygraph.

When oxidative phosphorylation was uncoupled from FCCP, embryos demonstrated a significantly higher rate of maximal mitochondrial respiration than larvae. This is most likely due to the fact that mitochondria in embryos are more active and abundant throughout embryonic development to provide energy for rapid cell division and differentiation, and mitochondrial activity may vary accordingly (Artuso et al., 2012; Arribat et al., 2019). This could lead to increased mitochondrial capacity in embryos. Embryos rely on the mother’s yolk sac, which may provide access to particular nutrients that promote mitochondrial respiration. We saw no changes in mitochondrion COX (complex IV) activity.

One striking result of this research project is the identification of sex differences in oxygen consumption. Even more surprising to us is the fact that in young adults (3-month-old) under minimal current, males on average have a two-fold increase in oxygen consumption compared to female. However, in older adults (12-month-old) female have a 30% increase in oxygen compared to males (Figure 3). Interestingly, the average oxygen consumption in a 3-month-old and a 12-month male is the same (160 pmol O_2_/min) (Figure 4A) while female show a 160% increase in oxygen consumption between 3 and 12 months of age (Figure 4B). We speculate that the dramatic increase in oxygen consumption in female is due to the energy consumed by the females during oocytes production. Zebrafish female start to produce viable oocyte at around 3 months but are at their peak of fecundity at around 12 months old (Hoo et al., 2016; Johnson et al., 2018). Female fish over the age of 12 months likely expend more energy on reproductive tasks such as egg production, driving an increased metabolic rate with increased mitochondrial oxygen consumption. Spermatozoid production requires less energy consumption for the male explaining while their oxygen consumption does not vary as the fish reach their sexual maturity (Mauch and Schoenwolf, 2001). Another difference observed due to sex differences in this report was the recovery in oxygen consumption after the adult zebrafish were subjected to physical activity (i.e., forced swimming in an increasing current, Figure 5). In our experimental settings it seems that the recovery in oxygen consumption is faster and greater in the females than in the males. While males display a steady decrease increase in oxygen consumption when subjected to a strong current (100 or 200 rpm), the decrease in oxygen consumption persist when the males are returned to the minimum current speed (26 rpm). However, in females although a slight decrease in oxygen consumption is observed at 100 rpm, all females tested show an increase in oxygen consumption when in a 200 rpm current but return rapidly to a normal level of oxygen consumption once put back in a 26 rpm current. The reason why female tends to recover better than males, at least in terms of oxygen consumption, when subjected to a physical effort remains elusive. However, zebrafish have a sexual dimorphism in term of size and weight with female being bigger and heavier than males (The zebrafish book). These anatomical differences in size and weight may explain why female after a physical effort can return rapidly to a normal level of oxygen consumption compared to males (Nüsslein-Volhard, 2012).

An interesting component of this study was the ability to measure oxidative phosphorylation cytochrome c oxidase (complex IV) activity in the whole zebrafish. Complex IV is a marker for the oxidative phosphorylation capacity (Larsen et al., 2012). However, there were limitations due to the limited access of TMPD to all of complex IV within the zebrafish. Typically, you would expect complex IV activity to be higher than basal activity if TMPD has complete access to all of the complex IV in the fish. In the future for a more accurate measure of complex IV activity, we will prepare zebrafish homogenates to compare age and sex. The homogenates can also be used to measure activities of other mitochondrial oxidative phosphorylation enzymes, markers for mitochondrial content such as citrate synthase (Larsen et al., 2012) and markers for oxidative damage such as aconitase activity (Yan et al., 1997). Data presented in this paper open the door to new physiological analysis in aquatic small species such as the zebrafish or medaka by allowing direct measurement of oxygen consumption of adult specimen under different water current speed. Future work using the Oroboros Oxygraph will certainly include the analysis of several mutant and/or transgenic fish lines with defects in mitochondria efficacy or metabolism.

## Data Availability

The original contributions presented in the study are included in the article/Supplementary Material, further inquiries can be directed to the corresponding author.

## Supplementary material

SUPPLEMENTAL FIGURE S1 Average basal respiration rates in young (3-month old) adult males (blue shaded bars) and females (red shaded bars) for data shown in Figure 5. Females are significantly lower than males. The respiration rate decreases more in males that females following physical activity. (** indicates a p value ≤ 0.01).
